# Artificial Intelligence Enhanced Electrocardiogram Analysis for Age and Sex Classification in Youth

**DOI:** 10.1007/s00246-025-04118-7

**Published:** 2026-02-18

**Authors:** Honggen Zhang, Mohammad Zaeri-Amirani, Mojtaba Abolfazli, Narayana P. Santhanam, June Zhang, Anders Høst-Madsen, Chieko Kimata, James C. Perry, Andras Bratincsak

**Affiliations:** 1 Department of Electrical and Computer Engineering, College of Engineering, University of Hawaii, Honolulu, HI USA; 2 Patient Safety & Quality Services, Hawaii Pacific Health, Honolulu, HI USA; 3 Departments of Pediatrics and Bioengineering, University of California San Diego, San Diego, CA USA; 4 Department of Pediatrics, John A. Burns School of Medicine, University of Hawaii, Honolulu, HI USA; 5 Hawaii Pacific Health Medical Group, Hawaii Pacific Health, 1319 Punahou St., Ste 950, Honolulu, HI 96826 USA

**Keywords:** Artificial intelligence, Machine learning, Electrocardiogram, Standards, Pediatric, Screening

## Abstract

Electrocardiogram (ECG) values vary significantly across age and sex, particularly during childhood and adolescence. While age- and sex-specific ECG standards exist, they often fail to capture complex multi-dimensional relationships and have not been applied in machine learning (ML) enhanced ECG analysis. Accuracy of automated ECG analysis in clinical practice improved significantly by applying ML models, however there is a paucity of such studies in the pediatric population. Our aim was to develop age- and sex-classification for children using ECG features with various ML models. We analyzed 29,408 curated resting 12-lead ECGs from healthy subjects aged 0–21 years using 177 digitized ECG variables combined with various ML models including regression and classification analyses and semi-supervised neural networks. Primary outcome variables were age and sex. Model performance was evaluated using F1-score, AUROC, and confusion matrices across repeated train-test splits. Support vector machine (SVM) achieved the highest accuracy in modeling both age and sex. Key predictive features included heart rate, PR interval, QRS duration, and T-wave amplitude. Age-group classification achieved an average true positive rate of 60% with SVM, improving to 94% when allowing one-group misclassification. Sex classification reached F1-scores of 0.91 and AUROC of 0.95 in adolescents and young adults, and moderate accuracy in younger children. Traditional supervised ML models can accurately model physiologic ECG changes related to age and sex, outperforming neural networks, particularly in smaller subgroups. These findings support the feasibility of ML models to capture of age- and sex-related ECG signatures to may aid future research and clinical applications in pediatric cardiology.

## Introduction

Since its invention by Einthoven in 1898, the electrocardiogram (ECG) has been one of the most important screening and diagnostic tests for heart problems [1] However, ECG analysis with the ability to indicate underlying cardiac disease states has historically relied upon the inherent biases and errors of physician interpretation based on standards derived from very few healthy subjects. In pediatric and adolescent populations, ECG interpretation is further hindered by the distinct challenges due to the rapid and profound physiological changes that occur from birth through young adulthood. These developmental changes affect ECG waveform morphology and timing, reflecting underlying alterations in heart size, autonomic tone, ion channel function, and conduction pathways. Detailed assessment of the largest historical pediatric cohort of normal ECGs recently allowed us to understand the granularity of these changes in the parameters of ECG variables across childhood [2]. Yet, these standards are primarily based on summary statistics of isolated ECG variables and do not fully capture the complex, multivariate patterns embedded within the high-dimensional ECG signal. Recognizing these dynamic and complex features is essential for accurate clinical interpretation. Recent advances in machine learning (ML) have ignited the development of a novel field: artificial intelligence (AI) enhanced ECG analysis [3–5]. These ML models offer the potential to leverage the full complexity of data in ECGs, and can integrate numerous ECG features simultaneously, uncovering latent patterns, and improving the granularity of physiological modeling. Importantly, establishing ML models that can accurately infer age and sex based solely on ECG data is a critical step toward developing adaptive, context-aware diagnostic tools. These models can identify subtle electrophysiological signatures correlated with demographic factors, thus providing a normative framework that enhances detection of pathological deviations. AI-enhanced ECG analysis has been used for the detection of ventricular dysfunction and hypertrophic cardiomyopathy [6–8] and was also able to model the age and sex of adults and estimate a biological or cardiac age of a person [9, 10]. However, these studies were performed in adults. There is a paucity of data of AI-enhanced ECG analysis in children, complicated by the profound changes in the ECG that occur between 0 and 21 years of age. There are several recent studies demonstrating accuracy of AI-enhanced ECG analysis in detecting hypertrophic cardiomyopathy (HCM), left ventricular dysfunction, congenitally corrected transposition of the great arteries, prolonged QT and Wolff-Parkinson-White syndrome [11–14], and even the determination of sex in children [15]. However, other less common heart conditions have not been modeled by AI in children. ECG analysis in children and youth has a unique importance in screening for rare heart conditions, congenital heart defects and inherited arrhythmia syndromes [16–19], but this screening cannot be performed in an automated model, if there is no definition of age-and sex-specific normal standards. To date, AI-generated age- and sex-specific ECG standards for children and adolescents have not been developed.

This study aims to evaluate multiple supervised and semi-supervised ML architectures to classify age groups and sex from ECG features of pediatric and young adult individuals. By developing age- and sex-classification, we aim to establish a foundation for automated AI-enhanced ECG analysis leveraging age- and sex-specific standards for children and young adults.

## Methods

### ECG Data Source and Study Population

This study used a large, curated cohort of ECGs from subjects with no known heart condition. The cohort, previously validated and published included ECGs collected retrospectively from patients aged 1 day to 21 years at Hawaii Pacific Health and Rady Children’s Hospital San Diego between 2012 and 2022 (*n* = 70,816) [2]. ECGs were obtained for a variety of clinical reasons, including evaluation of heart murmur, irregular heartbeat, syncope, dizziness, bradycardia, tachycardia, fever, screening for certain diseases, and for sports pre-participation screening. ECG from patients with congenital or acquired heart conditions, history of heart surgery, arrhythmia syndromes, pacemakers, or duplicate ECGs from the same patient within the same age-group were excluded from the study (*n* = 31,278). For multiple ECGs from the same patient within the same age group, we retained the earliest complete, highest-quality ECG, all other ECGs within the same age-group were excluded. ECGs from the same patient in different age groups were retained for the analysis and modeling of different age-groups. The stringent exclusion criteria created a curated cohort of ECGs from children and adolescents with no evidence of heart defect or cardiac anomaly on a more than 7-year average follow-up. The cohort was divided based on prior ECG age classification following the developmental stages of children and young adults, to the following 9 age-groups: (1) term newborns: 1–6 days old; (2) neonates: 1–4 weeks old; (3) young infants: 1 months to < 6 months old; (4) older infants: 6 months to < 2 years old; (5) toddlers and small children: 2 to < 5 years old; (6) children: 5 to < 9 years old; (7) preteen children: 9 to < 13 years old; (8) teenagers: 13 to < 17 years old; 9), and adolescents to young adults: 17 to < 22 years old. The number of patients in each age group ranged from 304 to 7,366 (Table 1).

**Table 1:** Number of study subjects from 1 day to 21 years sorted into 9 age-groups

Age groups	1	2	3	4	5	6	7	8	9
Ages	1–6 days	1–4 weeks	5 weeks − 5 months	6–23 months	2–4 years	5–8 years	9–12 years	13–16 years	17–21 years
N	304	684	1328	1766	2384	3075	4323	8178	7366
male	162	363	740	948	1227	1574	1896	3032	2376
female	142	321	588	818	1157	1501	2427	5146	4990

All ECGs were performed in resting supine position using GE MAC 5500 HD ECG systems (General Electrics, Houston, TX) at 500 Hz sampling frequency with standardized voltage (10 mm = 1 mV) and speed (25 mm/s). Digitized ECG values from all 12 leads were exported from the GE Muse v9 system without any pre-processing. ECGs were excluded from the final analysis if they had technical errors, such as lead reversal, poor baseline, or missing lead information (*n* = 10,130), resulting in a final cohort of 29,408 complete ECGs. The study was approved by the Hawaii Pacific Health Research Institute and deemed exempt from further Institutional Review Board approval due to the retrospective nature of the analysis.

### ECG Variable Selection and Processing

ECG variables were pre-selected based on expert physician input and prior established standards. We included 177 ECG variables (features) in the analysis of the ECGs, such as: P, QRS, and T axes; frontal QRS-T and spatial QRS-T angles, RR interval; PR interval; QRS duration; QT interval and corrected QT interval (QTc) calculated using the Bazett and Fridericia methods; peak amplitudes of P, Q, R, S and T waves; QRS integral; and T wave integral in all leads (I, II, III, aVL, aVF, aVR, V1, V2, V3, V4, V5, V6).

### Machine Learning Models, Training and Testing

Selected ML models were used on standardized digitized values of 177 ECG variables. We compared traditional ML models and neural networks for the determination of age and sex. Assessment with traditional ML models included Support Vector Machines (SVM) with linear and Radial Basis Function (RBF) kernels, Adaptive Boosting (AdaBoost) with decision trees, and Linear Discriminant Analysis (LDA) models. For neural nets we compared Triplets Bidirectional Generative Adversarial Networks (T-BiGAN) and Residual Networks (ResNet).

#### Traditional ML Models

Analysis with SVM is usually employed to solve complex classifications with a sensitive detection of outliers by defining boundaries among data points predetermined by certain supervised inputs. We used SVM in both the original data space with a linear kernel and in a new feature space obtained by a non-linear transformation of the data using RBF kernel. with penalty C = 0.5, 1, and 1.5 analyzed and optimal penalty C = 1.5 selected for age classification, and the default C = 1 for sex classification.

AdaBoost is a popular ensemble-based method for data classification that can enhance the power of a base/weak classifiers by a weighted linear combination of original data. We used AdaBoost with a decision tree model as the base estimator with hyperparameter depth of D = 1,3,5 tested, and learning rate L = 0.1, 1, 2 tested. The base estimator was set at 200 with the optimal hyperparameter combination of D = 5 and L = 1 for age classification, while for sex classification the base estimator was 50, D = 1, and L = 1.

LDA is a multi-class classification model that can be used for supervised learning by maximizing class separation on a low-dimensional space. We used LDA to separate multiple classes with multiple features based on data dimensionality reduction involving the entire ellipse of data and not only data on the boundary of distinct groups.

Age classification is a multi-class classification, and we used the entire dataset to develop the model. Model performance was evaluated by ratio of predicted and true labels for the determination of age-across the 0–21 years of the cohort. Sex classification is a binary determination. Since the difference in ECG variables is more pronounced among various ages than between the two sexes, we performed binary analysis to determine the sex of the subjects within each age-group. The performance of binary classification for SVM, AdaBoost and LDA was assessed using both the F1 score and receiver operating characteristic (ROC) curves as comparison metrics. We calculated ROC with the probability of each sample being assigned to one class. For model classification accuracy we used the F1-score metric, which combines precision and recall scores.

We performed continuous regression analysis across the entire age range and binary analysis using specific age-bins with distinct margins. We decided to emphasize results of age-classification using the distinct age-bins following previous physiological classification of infants and children into distinct age groups, because a confidence interval and error margin of ± 1.2 years was deemed to be too large and meaningless, when we had to compare 1–6 days old and 2–4 weeks old infants. Nevertheless, we included both results in our analysis, and presented the results of both the regression analysis and the multi-class analysis.

#### Neural Networks

T-BiGAN is a ML model that offers improved feature representation through semi-supervised learning, employing a model based on Bidirectional Generative Adversarial Networks (BiGAN). In this approach, semi-supervised data is seamlessly integrated into the training process via an additional triplet loss term. The BiGAN structure comprises an encoder and a decoder, facilitating data transformation into a latent space, alongside a discriminator tasked with distinguishing genuine data from synthetic data within the GAN framework. In T-BiGAN, auxiliary labels within the dataset are utilized. The choice of triplet loss is deliberated: during training, the model considers a probability where the distance from a query example to a negative example (i.e. those with labels different from the query) should be greater than the distance to positive examples. This strategic use of triplet loss fosters a mapping in the latent space that encourage data with the same label to form distinct clusters, differentiating them from data with other labels. This is the underlying rationale for the application of T-BiGAN on ECG data.

In our T-BiGAN model, we incorporated sex and age groups (2 sex-groups × 9 age-groups = 18 categories) as auxiliary labels during the training process. The model uses a latent dimension of 50 z_dim = 50), and is comprised of three main components: an encoder, a generator, and a discriminator. Each of these neural components consists of two hidden layers, with each layer having a size of 1024 units followed by a leaky Rectified Linear Unit (ReLU) activation layer with a negative slope of 0.2 for non-linearity. Additionally, the layers in the generator are followed by Batch Normalization layers. The optimizer is Adam with a very small initial learning rate of 1e−8 and β_1_ = 0.5, making the training stable but very slow to start. The model is regularized using L2 weight decay (2.5e−5) and weights are initialized with a small Gaussian noise (stddev = 0.02). Training is run for 501 epochs with a batch size of 256.

Residual Networks (ResNet) is a deep neural network architecture designed to facilitate the training of exceptionally deep networks. It achieves this by introducing residual blocks, which utilize skip connections to learn the difference (residual) between input and desired output in each block. ResNet architectures often incorporate batch normalization, global average pooling, and some in various depths.

The ResNet model comprises of an initial convolutional layer that applies 32 filters, batch normalization, ReLU activation, and max-pooling. The ResNet-based classifier is built on top of ImageNet-pretrained ResNet-50, using it as a feature extractor (with all layers frozen). The output of the ResNet backbone is passed through a custom fully connected head, consisting of dense layers of sizes 512, 256, 128, and 64, each followed by ReLU activation and 50% dropout to prevent overfitting. The final layer is a Softmax classifier that outputs class probabilities. The model is compiled with the Adam optimizer, using categorical cross-entropy loss and accuracy as a metric. We understand that a neural net pretrained on ECG features would improve the performance of prediction. We used this model as a simple comparative assessment of an untrained neural network.

Similarly to the traditional ML models, T-BiGAN and ResNet models were used to characterize the sex of the subjects in a binary classification, while the age-group classification used a multi-class model for the determination of set age-groups.

### Training and Testing

For all traditional ML models (SVM, AdaBoost, LDA) and neural nets (T-BiGAN, ResNet) 75% of data was used for training, 10% for testing and hyperparameter tuning, and 15% for final classification. To minimize overfitting and report performance variance, we used k-fold cross-validation.

### Statistical Analysis

For binary classification, simple descriptive statistical measures were calculated (true positive, false positive, true negative, and false negative rates). From those rates, ROC curves were generated and Area Under the ROC Curve (AUROC) was calculated. For predictive accuracy, precision rates or positive predictive values (true positive divided by the sum of true and false positive), and recall or sensitivity (true positive divided by the sum of true positive and false negative) rates were calculated, next F1 scores were generated as the harmonic mean of the precision and recall rates, being one of the most accurate measures of test predictability. For multiple group classification confusion matrices were generated to assess true and predicted positive rates. For regression analysis, we modeled performance as a continuous function of age using a natural cubic spline. For each model outcome we fit an ordinary least-squares regression with spline basis functions (df = 5), which allowed a monotonic-by-segment curve representation of the entire cohort. The fitted model was evaluated on a dense grid of ages (0–21 years) to obtain the performance curves with 95% confidence bands.

## Results

### Study Population

After exclusion of patients with heart conditions, duplicate and erroneous ECGs, the final study cohort contained 29,408 curated normal ECGs across ages of 1 day to 21 years (12,318 male—41.9%, 17,090 female—58.1%) (Table 1).

### Modeling of Sex in Various Ages

**Fig. 1:**
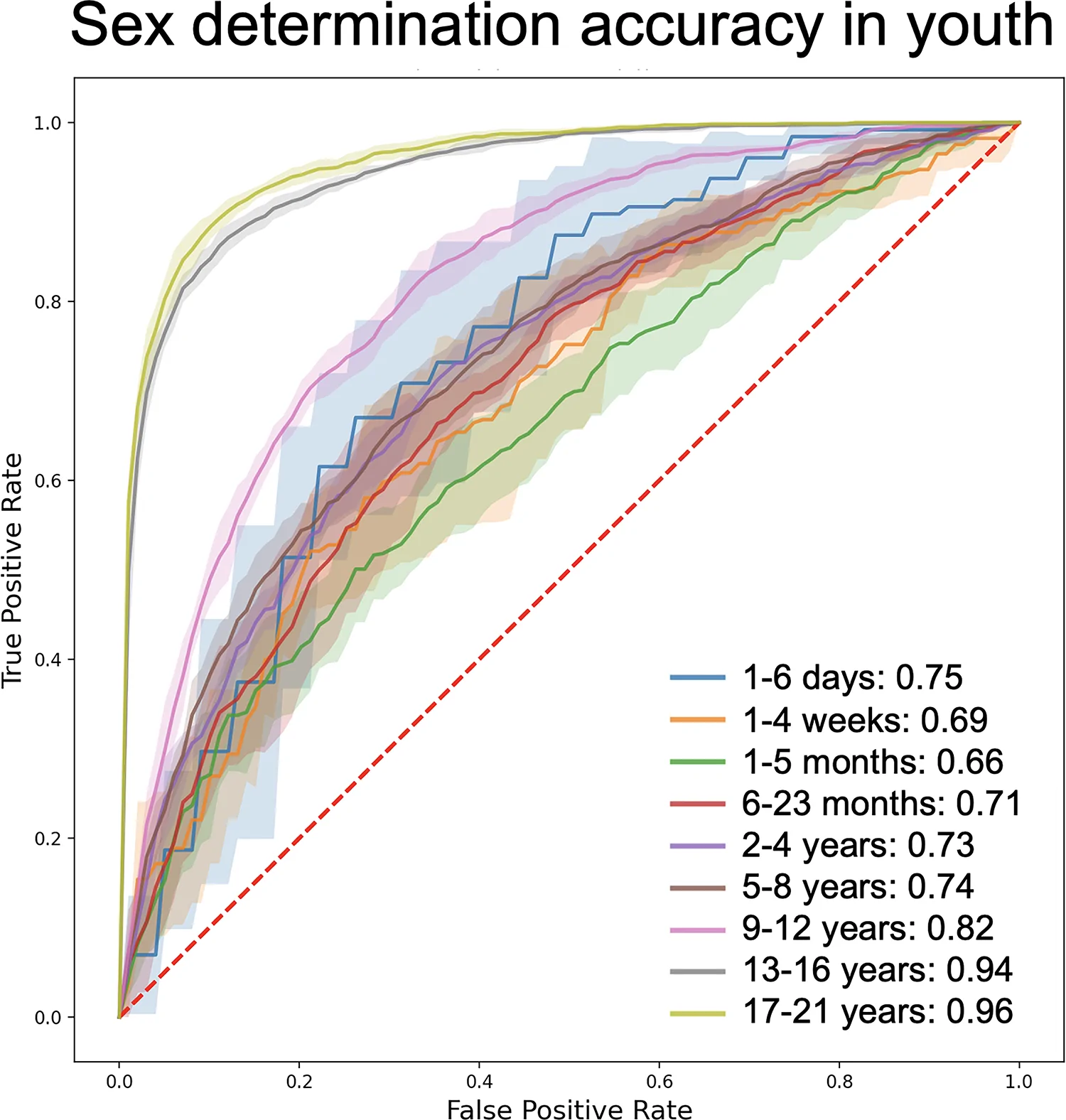
Sex determination accuracy in youth: Classification of sex in various age groups displayed as Receiver Operating Characteristic curves using Support Vector Machine supervised machine learning model on 12-lead electrocardiograms. Sex could not be distinguished with high accuracy in age groups 1–6: ages 1 day to 8 years, consistent with minimal difference in the somatic appearance of girls and boys. Sex was classified with acceptable accuracy (82% Area Under the Curve) in age group 7: ages 9 years to 12 years, in the pre-pubertal age. Sex was predicted with very high accuracy (94–95% AUROC) in age groups 8–9: in ages 13 years and older, consistent with somatic differences between males and females. Age groups 1: 1–6 days old; 2: 1–4 weeks old; 3: 1 months to < 6 months old; 4: 6 months to < 2 years old; 5: 2 to < 5 years old; 6: 5 to < 9 years old; 7: 9 to < 13 years old; 8: 13 to < 17 years old; 9: 17 to < 22 years old

**Fig. 2:**
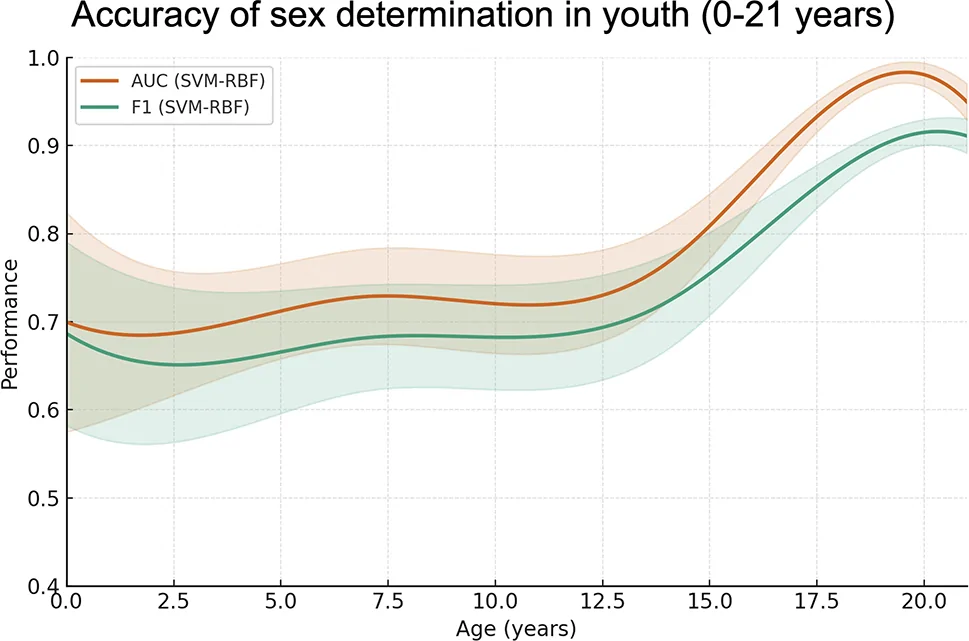
Continuous curve showing sex-classification performance (SVM-RBF) across the pediatric age range. Changes in the AUROC (orange) and F1 score (green) from birth to 21 years. Performance is modest in infancy and early childhood, rises gradually through school age, and increases sharply in adolescence, plateauing at high accuracy in teenagers and young adults

**Fig. 3:**
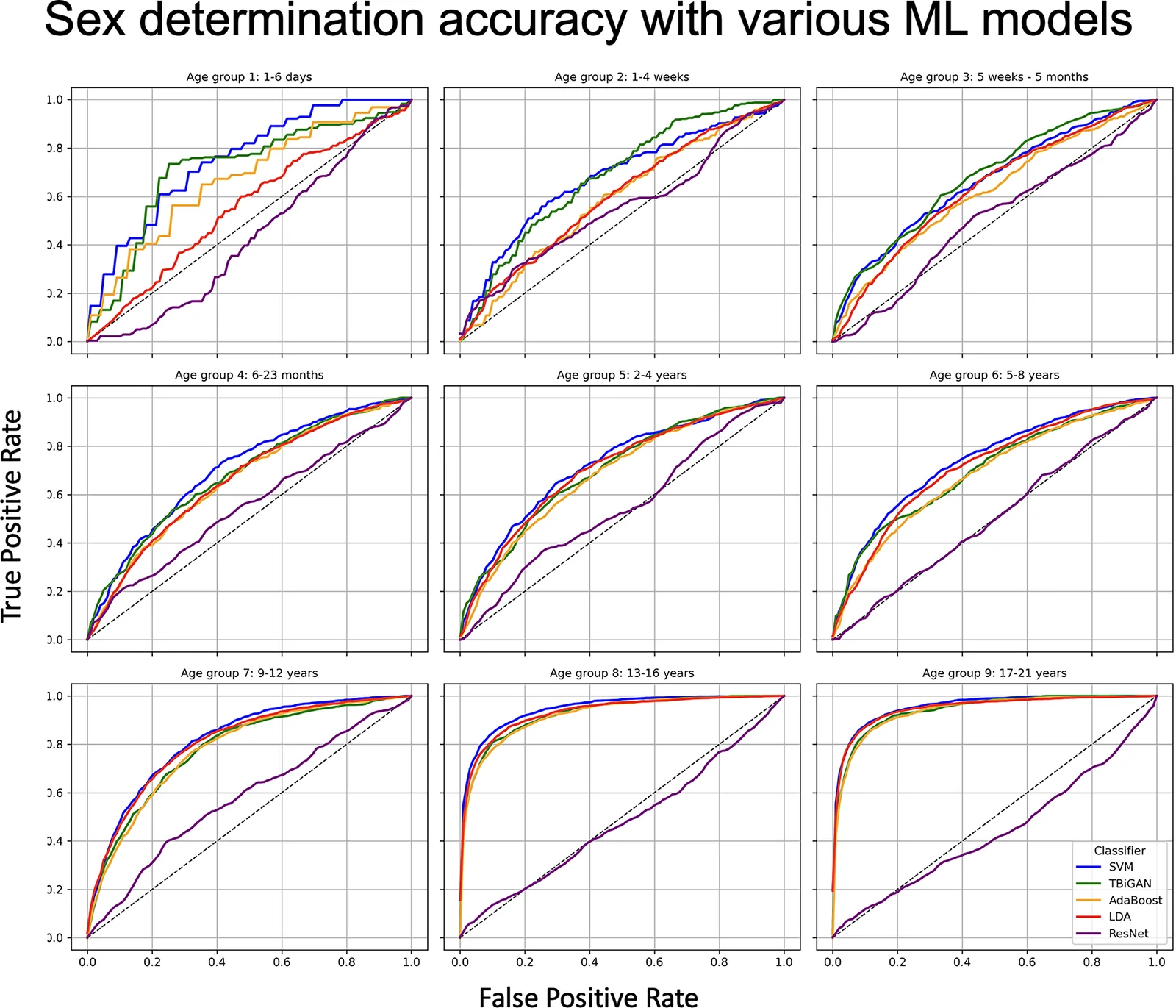
Sex determination accuracy with various ML models: Comparison of various machine learning models in the classification of sex across various ages using selected ML models, such as Support Vector Machines (SVM) with Radial Basis Function (RBF) kernels, Adaptive Boosting (AdaBoost) with decision trees, Linear Discriminant Analysis (LDA), Triplets Bidirectional Generative Adversarial Networks (T-BiGAN) and Residual Networks (ResNet), demonstrated that traditional models (SVM, AdaBoost, LDA) outperformed neural networks in the classification of sex in every age-group. SVM with RBF kernel resulted in the highest prediction accuracy. All ML models showed higher accuracy in older age-groups, ages 13 years and older

Sex-related differences in ECG parameters were subtle and often masked by the more pronounced age-related changes. To control for the age-related differences, sex classification models were trained and tested separately within each age group. Predictive accuracy varied by age group and the ML model used. The summated area under the ROC curve in distinguishing male and female ECGs ranged from 61% in younger children to 96% in teenagers and young adults (Fig. 1; Table 2). Consistent with the AUROC scores, F1 scores for determining sex ranged from 0.53 to 0.91, depending on the age group and the ML model used (Table 2). Accuracy of sex prediction was considerable in the newborn period, decreased in infancy and school age children, improved in preteen children and became excellent in teenagers and young adults (Fig. 2). The best sex classification performance was achieved in teenagers (13–17 years) and young adults (18–21 years) with an AUROC pf 95% and an F1 score of 0.91, compared to AUROC of 65–82% and F1 scores of 0.53–0.7 in younger children (0–12 years) (Fig. 1; Table 2). Traditional ML models, such as SVM, consistently outperformed neural networks, such as ResNet, in predicting sex along all age groups, e.g. an F1 score of 0.91 by SVM vs. 0.44 by ResNet, and an AUROC of 96% by SVM vs. 47% by ResNet in the 17–21 years old group (age group 9) (Fig. 3; Table 2).

**Table 2:** Area under the receiver operating characteristic curve and F1 accuracy scores in differentiating sex using various machine learning models and 5-fold cross-validation

	Area under the receiver operating characteristic curves								
Age groups	1	2	3	4	5	6	7	8	9
SVM-RBF	0.75 ± 0.12	0.69 ± 0.12	0.66 ± 0.08	0.71 ± 0.05	0.73 ± 0.05	0.74 ± 0.06	0.82 ± 0.03	0.94 ± 0.02	0.96 ± 0.02
AdaBoost	0.76 ± 0.13	0.66 ± 0.08	0.66 ± 0.05	0.72 ± 0.06	0.73 ± 0.09	0.75 ± 0.05	0.82 ± 0.05	0.94 ± 0.02	0.95 ± 0.01
LDA	0.55 ± 0.04	0.60 ± 0.03	0.64 ± 0.03	0.66 ± 0.01	0.71 ± 0.01	0.72 ± 0.01	0.81 ± 0.01	0.93 ± 0.01	0.95 ± 0.01
T-BiGAN	0.72 ± 0.04	0.68 ± 0.03	0.68 ± 0.02	0.68 ± 0.02	0.70 ± 0.01	0.70 ± 0.01	0.78 ± 0.01	0.93 ± 0.01	0.94 ± 0.01
Resnet	0.42 ± 0.04	0.55 ± 0.03	0.51 ± 0.02	0.52 ± 0.02	0.52 ± 0.01	0.50 ± 0.01	0.56 ± 0.01	0.49 ± 0.01	0.47 ± 0.01

	F1 accuracy scores								
Age groups	1	2	3	4	5	6	7	8	9
SVM-RBF	0.70 ± 0.1	0.66 ± 0.11	0.65 ± 0.09	0.65 ± 0.08	0.68 ± 0.06	0.71 ± 0.05	0.75 ± 0.05	0.87 ± 0.02	0.91 ± 0.02
AdaBoost	0.66 ± 0.12	0.61 ± 0.11	0.60 ± 0.08	0.63 ± 0.07	0.64 ± 0.06	0.67 ± 0.05	0.70 ± 0.04	0.85 ± 0.02	0.89 ± 0.01
LDA	0.53 ± 0.03	0.63 ± 0.03	0.61 ± 0.03	0.60 ± 0.02	0.63 ± 0.01	0.63 ± 0.01	0.71 ± 0.01	0.83 ± 0.01	0.83 ± 0.01
T-BiGAN	0.61 ± 0.04	0.60 ± 0.03	0.57 ± 0.02	0.61 ± 0.02	0.64 ± 0.01	0.64 ± 0.01	0.71 ± 0.01	0.85 ± 0.01	0.86 ± 0.01
Resnet	0.41 ± 0.04	0.55 ± 0.03	0.52 ± 0.02	0.51 ± 0.02	0.53 ± 0.01	0.51 ± 0.01	0.58 ± 0.01	0.51 ± 0.01	0.44 ± 0.01

Age groups: 1: 1–6 days old; 2: 1–4 weeks old; 3: 1 months to < 6 months old; 4: 6 months to < 2 years old; 5: 2 to < 5 years old; 6: 5 to < 9 years old; 7: 9 to < 13 years old; 8: 13 to < 17 years old; 9: 17 to < 22 years old; 10: 22 to < 30 years old; and 11: 30–40 years old. Machine learning models: Support Vector Machines (SVM) with Radial Basis Function (RBF) kernels, Adaptive Boosting (AdaBoost) with decision trees, Linear Discriminant Analysis (LDA), Triplets Bidirectional Generative Adversarial Networks (T-BiGAN) and Residual Networks (ResNet)

### Modeling of Age from Infancy to Young Adulthood

**Fig. 4:**
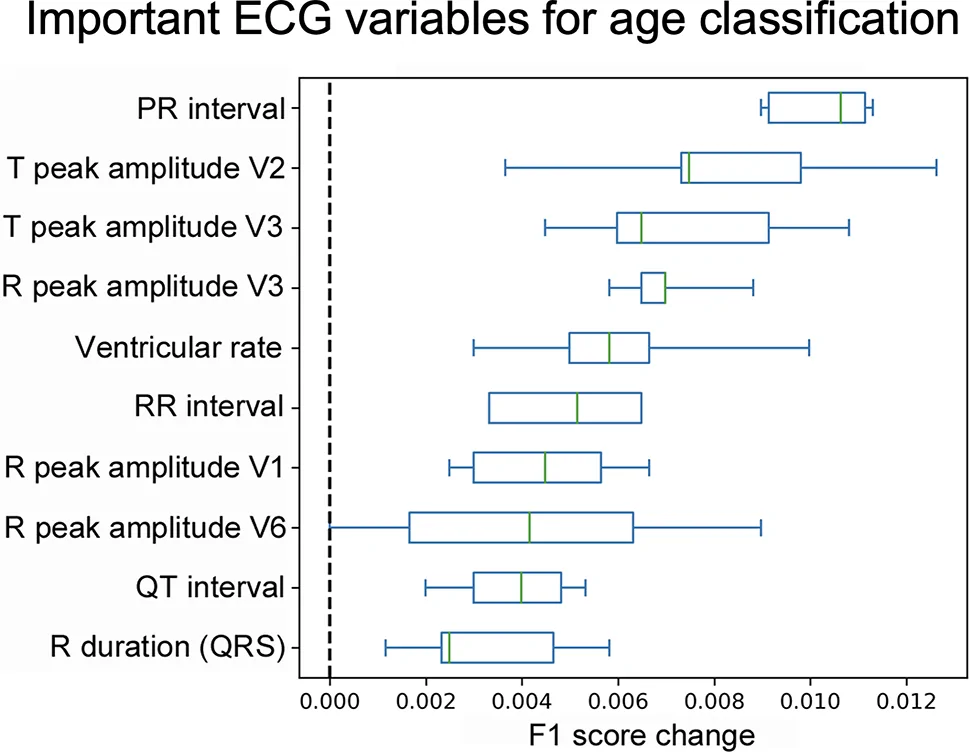
Important ECG features for age classification: Certain ECG variables had a high impact on age classification. These variables, such as PR, RR and QT intervals, QRS duration, R and T wave amplitudes in precordial leads, appeared to be more important for the determination of age by supervised machine learning models demonstrated by the impact of F1 accuracy score changes

**Fig. 5:**
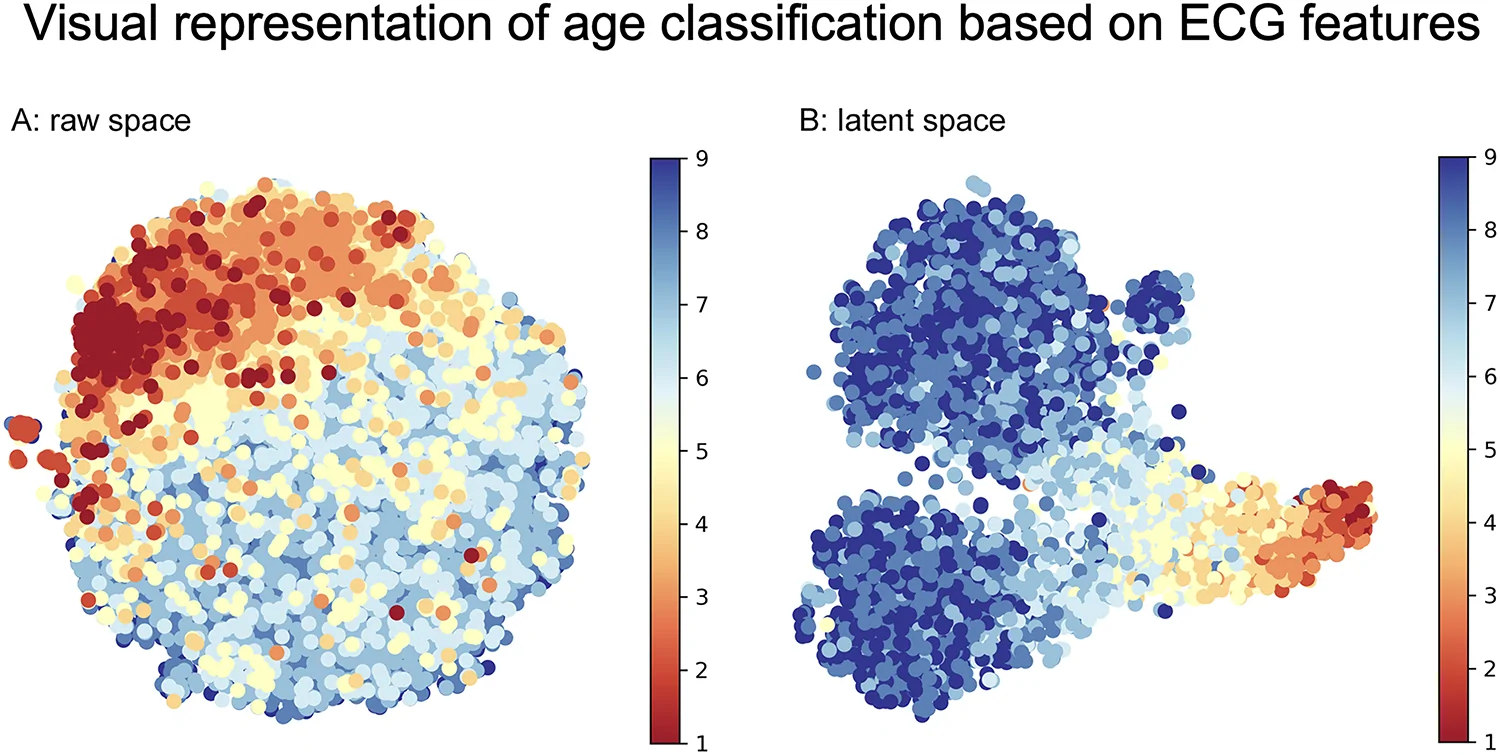
Visual representation of age classification based on ECG features: Two-dimensional display of the age-distribution of 29,408 children and adolescents (ages 1 day to 21 years, age group 1—red, age-group 9—blue) compares a simple regression analysis in raw space (**A**) and after translation into latent space (**B**) using Bidirectional Generalized Adversarial Network semi-supervised machine learning model. Distribution in latent space created a higher resolution and better prediction of age compared to simple regression analysis

Numerous ECG variables showed variations among different age-groups in children and young adults. The following ECG features were identified having a higher importance using the permutation importance method: heart rate, PR interval, QRS duration, QTc interval, and R and T wave amplitudes in V1, V3 and V6 (Fig. 4). These key features were explicitly utilized in the supervised ML models to develop age-group prediction, while the semi-supervised T-BiGAN model processed all features without any discrimination or weight, and transformed to latent space to develop age-group classification. Visual representation of individual data points reflects how the accuracy of age prediction improved when the data was transformed to latent space (Fig. 5).

**Fig. 6:**
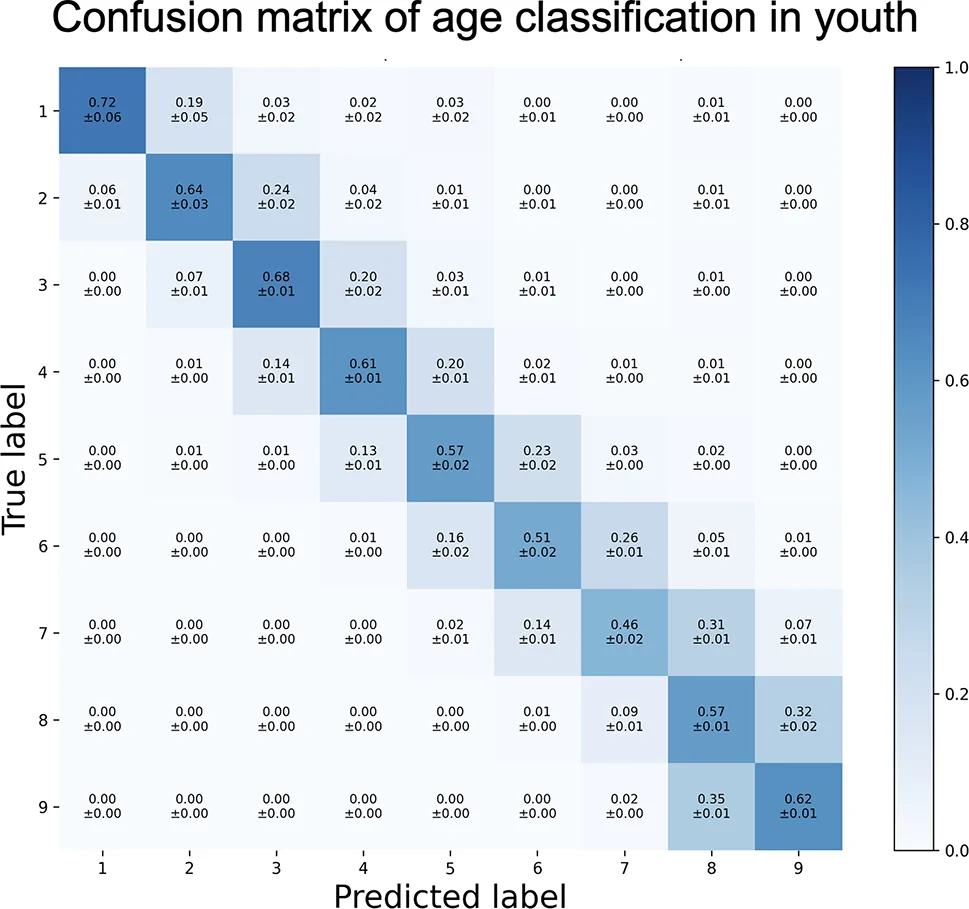
Confusion matrix of age classification in youth: Accuracy of age classification displayed as a confusion matrix in various age groups using Support Vector Machine supervised machine learning model. Age could be classified with high accuracy in the youngest age groups (age groups 1–3, ages 1 day to 5 months) with true positive rates (TPRs) of 64–72%, compared to older children (age groups 4–6, ages 6 months to 8 years) with TPRs of 51–61%, and adolescents and young adults (age groups 7–9, ages 13–21 years) with TPRs of 46–62%. Adjusting the analysis allowing ± 1 age-group deviation improved the TPR to 91–99% in all ages. Age groups 1: 1–6 days old; 2: 1–4 weeks old; 3: 1 months to < 6 months old; 4: 6 months to < 2 years old; 5: 2 to < 5 years old; 6: 5 to < 9 years old; 7: 9 to < 13 years old; 8: 13 to < 17 years old; 9: 17 to < 22 years old

All models successfully discriminated among the predefined 9 age-groups in the multi-class classification model, with a true positive rate (TPR) ranging from 46 to 72%. The highest accuracy was observed in distinguishing the youngest age groups (1–6 days, 1–4 weeks, 1–5 months old) with TPRs of 64–72%, compared to older age groups (2–21 years), with TPRs of 46–62% (Fig. 6). Traditional supervised models performed better than neural networks. SVM with RBF kernel showed the highest accuracy with an average TPR of 60%, outperforming AdaBoost (49%), and LDA (55%). Younger age-groups with smaller number of subjects had a higher variation of results depending on hyperparameter optimization, reflected by wider confidence intervals, nevertheless, SVM outperformed the other models in multi-class classification despite hyperparameter optimization. Among neural networks, T-BiGAN had the highest average TPR (57%) in multi-class classification of various ages compared to Resnet (39%). In addition to multi-class classification, regression analysis across the entire studied age-range showed a consistently high predication accuracy of age with a confidence interval of ± 1.2 years. This might be appropriate in older ages, but becomes less meaningful in distinguishing newborns, infants and toddlers of 0–2 years of age.

**Fig. 7:**
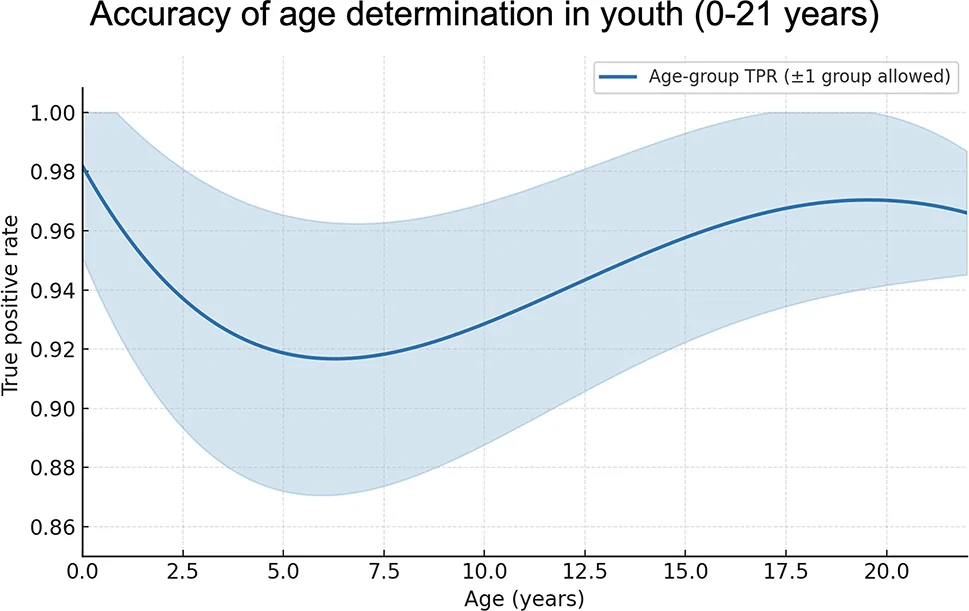
Continuous regression curve for age-group classification when a prediction within ± 1 adjacent age group was counted as correct. The curve shows uniformly high performance (≈ 0.91–0.99) across all ages, with a slightly lower region in mid-childhood and a smooth plateau in late adolescence. Shaded area indicates the variability (confidence interval) from the original bin-wise results

A confusion matrix visually represents the true positive rates using SVM with RBF kernel (Fig. 6). The matrix revealed that misclassification (false negative rate: FNR) predominantly occurred in immediate neighboring age-groups, consistent with the expected overlap in physiological changes. Allowing a single age-group deviation (± 1 age-group error margin), increased the average adjusted age-group detection accuracy (TPR) to 94% for SVM (range 91–99%), and 92% with T-BiGAN (range 88–98%), and a FNR of 1–9% with SVM, and 2–12% with T-BiGAN. A visual representation of the age-prediction across the entire age-range reflected high prediction accuracy (Fig. 7).

### Comparison of Various Machine Learning Models

Comparison of traditional supervised ML methods (SVM, AdaBoost, LDA) compared to neural networks (ResNet, T-BiGAN) demonstrated that when including less than 300 ECGs, traditional ML models (SVM with linear or RBF kernel) outperformed neural networks in predicting both age and sex (Fig. 3). When the analyzed data included 1000 or more ECGs, traditional models and neural nets had similar accuracy. Overall, the best results were achieved using SVM with RBF kernel in both sex-prediction with a binary classification, and age-prediction using a multi-class classification (Fig. 3).

## Discussion

Our findings demonstrate that machine learning models can accurately classify both age and sex from ECG features in children and young adults (Figs. 2 and 7). Notably, classification of age was achieved with high precision even in younger age-groups containing only a few hundred ECGs—previously not demonstrated with any other method (Figs. 6, 7). Our results support the concept that age- and sex-related physiological differences are encoded in the ECG waveform and can be decoded through data-driven methods. Modeling demographic-specific normal ECGs using specific ML models may facilitate the development of more precise, automated ECG interpretation frameworks.

### Age and Sex Classification

In early childhood, physiologic differences between males and females are minimal, but become more apparent around (10–12 years). Our results demonstrate that among adolescents and young adults, when males and females differ in physiologic features, their ECG also changes, reflected by subtle sex-related differences in specific parameters, such as PR interval, QRS duration, QTc interval, and R and S wave voltages in many leads. ML models detected these changes with high accuracy in adolescents and young adults, with AUROC values of 0.94–0.95. Such a high AUROC would serve as a remarkable metric for a screening tool, supporting the idea that the ECG encodes biologically relevant sex-specific signatures.

Similar to sex, age can be determined accurately by ECG in children and young adults. Somatic and physiologic changes occur during the development of children, with the most dramatic changes observed during early childhood (0–5 years), and less pronounced differences in adolescents and young adults (15 to 21 years). Following somatic growth, ECG variables change throughout childhood, with the most dramatic changes in early childhood, detected by specific ECG parameters, such as heart rate, PR, QRS and QTc intervals, and R and T wave voltages. Despite sample size limitations and class imbalance among analyzed groups, both supervised and semi-supervised ML models classified various ages with an excellent adjusted true positive rate of 88–99% by the ECG. This is the first ML-based multi-class model to classify nine distinct pediatric age groups using ECG data alone. Previous model showed similar performance in pre- and post-pubertal adolescents [15]. The proof of the concept of multi-class classification using ML-enhanced ECG analysis is foundational in the process of developing an automated system for ECG analysis and defining normal ECGs for every age and sex.

### Machine Learning Model Comparison

Traditional ML models (SVM and AdaBoost) performed more favorably in prediction modeling of datasets with less than 1000 data points (ECGs) compared to neural networks (T-BiGAN and ResNet). Supervised and weighted preselection of ECG variables improved the prediction accuracy of traditional ML models. When a dataset (specific age group) contained more than 1000 data points (ECGs), the difference in performance between the traditional models and neural networks diminished. While previous ECG-based AI models relied on datasets in excess of 5,000 subjects, our study shows that effective classification and prediction is possible with much smaller subject numbers. Developing ML algorithms for ECG analysis that can model analyzed groups with less than a 1000 subjects, and distinguish these groups with remarkable accuracy is particularly relevant for rare cardiac disorders, where large datasets are not available.

### Clinical Significance of Sex and Age Classification in Youth

Our results highlight that ECG signatures differ by demographic group and that ML can reliably recognize these patterns. The most significantly affected ECG variables and ML modeling features were PR, QRS and QTc intervals and the R, S and T wave amplitudes. These very same ECG parameters are used to diagnose several heart conditions, including conduction defects, long QT syndrome (LQTS), and ventricular hypertrophy or enlargement associated with cardiomyopathies. The subtle changes in ECG variables of these heart conditions are not only affected, but could be easily masked by the changes caused by different age-groups and sexes. Without age- and sex-specific reference values, such abnormalities can be overlooked or misinterpreted. For example, a mildly prolonged QT interval may be normal in a 1-week-old female, but would serve as a suspected diagnosis in a 10-year-old male, and similarly a certain QRS duration and S wave amplitude in lead V2 could be normal in an 8-year-old female, but would be associated with significant ventricular enlargement or hypertrophy in a 2-year-old female.

Establishing robust normative ECG standards is crucial for future ML-based models aimed at detecting heart conditions in children. These models can only perform accurately if trained on well-defined demographic baselines with curated normal ECGs. By modeling healthy ECG profiles across pediatric and young adult age groups and sexes, we set the stage for ML tools that can provide demographic-specific diagnosis in clinical practice. It is important to emphasize that the primary aim of our study was not to predict age or sex as diagnostic endpoints, but rather to assess and model the representation of these factors in the ECG data. This distinction is important because accurate identification of age and sex signatures is a prerequisite for developing reliable diagnostic models that avoid confounding by demographic variability. In clinical practice, age and sex are known and recorded variables, but understanding their explicit ECG correlates enhances model transparency and interpretability, will serve as the foundation for future age-and sex-specific ML models for cardiac disease prediction, and provide an important step towards creating AI-enhanced ECG screening.

### Limitations and Strengths

Our study had certain limitations. Sex was self-reported, which may not always align with biologic sex—potentially affecting ECG patterns. Our ML analysis was limited to selected models, and excluded certain advanced AI methods, such as transformers and large language models, however, we believe that we have chosen representative models for comparison, and the exclusion of certain models reflected input signal and feature complexity.

Strengths of our study include the use of a curated dataset containing only healthy subjects, eliminating patients with cardiac conditions, and enabling the establishment of normal ECG values for accurate ML modeling. We also compared multiple ML algorithms, rather than relying on a single model, which strengthens confidence in our results and helps mitigate bias and overfitting. Achieving consistent findings across different methods suggests robustness of our findings. An additional limitation, nevertheless, is the lack of validation of our results with an external cohort.

## Conclusion

In conclusion, this study provides evidence that ML can uncover age- and sex-specific signatures in pediatric ECG data, even with smaller sample size. By establishing reliable age- and sex-specific ECG standards, this work could be used for future efforts to build ML models capable of identifying conditions with subtle ECG changes affected by age- and sex-specific variations. Our findings may improve the field of AI-ECG detection of rare conditions beyond static reference standards toward dynamic, ML-informed models that better capture biological variability and potentially aid in developing a personalized, context-aware ECG interpretation. Such AI-enhanced ECG analytic models incorporating demographic variation are poised to improve the accuracy and reliability of ECG interpretation, particularly for rare cardiac conditions in children, where sample sizes are limited and demographic variability is large.

## Data Availability

The datasets of electrocardiogram variables for children and young adults will be made partially available upon request. Due to patients’ privacy and regulations about HIPAA protected personal identifiable information, the entire dataset is not available as an open source and will not be made available, because patient-specific ECG parameters may identify an individual patient.

## References

[1] Fisch C (2000) Centennial of the string galvanometer and the electrocardiogram. J Am Coll Cardiol 36:1737–1745. 10.1016/s0735-1097(00)00976-1 11092639

[2] Bratincsák A, Kimata C, Limm-Chan BN, Vincent KP, Williams MR, Perry JC (2020) Electrocardiogram standards for children and young adults using Z-scores. Circ Arrhythm Electrophysiol 13:e008253. 10.1161/CIRCEP.119.008253 32634327

[3] Feeny A, Chung MK, Madabhushi A, Attia ZI, Cikes M, Firouznia M et al (2019) Artificial intelligence and machine learning in arrhythmias and electrophysiology. Circ Arrhythm Electrophysiol 12:e007952. 10.1161/CIRCEP.119.007952 PMC7808396 32628863

[4] Ribeiro AH, Ribeiro MH, Paixão GMM, Oliveira DM, Gomes PR, Canazart JA et al (2020) Automatic diagnosis of the 12-lead ECG using a deep neural network. Nat Commun 11:1760. 10.1038/s41467-020-15656-0 PMC7145824 32273514

[5] Siontis KC, Attia ZI, Friedman PA, Noseworthy PA, Kapa S, Lopez-Jimenez F et al (2021) Artificial intelligence-enhanced electrocardiography in cardiovascular disease management. Nat Rev Cardiol 18:349–360. 10.1038/s41569-020-00503-2 PMC7848866 33526938

[6] Attia ZI, Noseworthy PA, Lopez-Jimenez F, Asirvatham SJ, Deshmukh AJ, Gersh BJ et al (2019) An artificial intelligence-enabled ECG algorithm for the identification of patients with atrial fibrillation during sinus rhythm. Lancet 394:861–867. 10.1016/S0140-6736(19)31721-0 31378392

[7] Attia ZI, Kapa S, Lopez-Jimenez F, McKie PM, Ladewig DJ, Satam G et al (2019) Screening for cardiac contractile dysfunction using an artificial intelligence-enabled electrocardiogram. Nat Med 25:70–74. 10.1038/s41591-018-0240-2 30617318

[8] Ko WY, Siontis KC, Attia ZI, Carter RE, Kapa S, Ommen SR et al (2020) Detection of hypertrophic cardiomyopathy using a convolutional neural network-enabled electrocardiogram. J Am Coll Cardiol 75:722–733. 10.1016/j.jacc.2019.12.030 32081280

[9] Attia ZI, Friedman PA, Noseworthy PA, Lopez-Jimenez F, Ladewig DJ, Satam G et al (2019c) Age and sex prediction using an artificial intelligence-enabled electrocardiogram. Circ Arrhythm Electrophysiol 12:e007284. 10.1161/CIRCEP.119.007284 PMC7661045 31450977

[10] van der Wall EE (2022) International criteria for electrocardiographic interpretation in athletes: consensus statement. J Electrocardiol 71:1–6. 10.1016/j.jelectrocard.2021.12.001 28258178

[11] Siontis KC, Noseworthy PA, Attia ZI, Carter RE, Yao X, Kapa S et al (2021) Detection of hypertrophic cardiomyopathy by an artificial intelligence electrocardiogram in children and adolescents. Int J Cardiol 340:42–47. 10.1016/j.ijcard.2021.08.026 34419527

[12] Mayourian J, Kaye D, Chowdhury D, Konerman M, Moghaddam AN, Sambidi P et al (2024) Pediatric ECG-based deep learning to predict left ventricular dysfunction and remodeling. Circulation 149:917–931. 10.1161/CIRCULATIONAHA.123.067750 PMC10948312 38314583

[13] Ghelani SJ, Thatte N, La Cava W, Triedman JK, Mayourian J (2025) Artificial Intelligence-Enabled ECG to detect congenitally corrected transposition of the great arteries. Pediatr Cardiol. 10.1007/s00246-025-03916-3Epub ahead of print. PMID: 40523997 40523997

[14] Mayourian J, La Cava WG, de Ferranti SD, Mah D, Alexander M, Walsh E, Triedman JK (2025) Expert-level automated diagnosis of the pediatric ECG using a deep neural network. JACC Clin Electrophysiol 11(6):1308–1320. 10.1016/j.jacep.2025.02.003 PMC12197845 40100196

[15] O’Sullivan D, Anjewierden S, Greason G, Attia IZ, Lopez-Jimenez F, Friedman PA et al (2024) Pediatric sex estimation using AI-enabled ECG analysis: influence of pubertal development. NPJ Digit Med 7(1):176. 10.1038/s41746-024-01165-x PMC11220019 38956410

[16] Gillette PC, Garson A (1992) Sudden cardiac death in the pediatric population. Circulation 85:I64–I69. 10.1161/01.CIR.85.1_suppl.I64 1728507

[17] Rodday AM, Tryka KA, King ME, Goodwin J, Graham D, Parsons SK (2012) Electrocardiogram screening for disorders that cause sudden cardiac death in asymptomatic children: a meta-analysis. Pediatrics 129:e999–e1010. 10.1542/peds.2011-0643 PMC3313631 22392183

[18] Drezner JA, Ackerman MJ, Anderson J, Ashley E, Asplund CA, Baggish AL et al (2017) International criteria for electrocardiographic interpretation in athletes: consensus statement. Br J Sports Med 51:704–731. 10.1136/bjsports-2016-097331 28258178

[19] Sarto P, Zorzi A, Merlo L, Cerrone M, Cipriani A, Mattioli AV et al (2023) Value of screening for the risk of sudden cardiac death in young competitive athletes. Eur Heart J 44:1084–1092. 10.1093/eurheartj/ehac015 PMC10027466 36760222

